# Oral Metformin Treatment Counteracts Adipoinsular Axis Dysfunction in Hypothalamic Obese Rats

**DOI:** 10.1155/2015/284042

**Published:** 2015-04-05

**Authors:** Daniel Castrogiovanni, Luisina Ongaro, Guillermina Zuburía, Andrés Giovambattista, Eduardo Spinedi

**Affiliations:** ^1^Neuroendocrine Unit, IMBICE (CONICET-CICPBA), La Plata Medical School, 1900 La Plata, Argentina; ^2^CENEXA (UNLP-CONICET La Plata), La Plata Medical School, 1900 La Plata, Argentina

## Abstract

Rats neonatally treated with monosodium L-glutamate (MSG) are deeply dysfunctional in adulthood. We explored the effect of an oral low dose of metformin treatment in male MSG rats on adipoinsular axis and visceral adipose tissue (VAT) dysfunctions, in both basal (nonfasting) and endotoxemia conditions. MSG rats, treated or not treated with metformin (30 days prior to experimentation), and control litter-mates (CTR) were studied at 90 days of age. Peripheral concentrations of glucose, lipids, and hormones were determined in basal and post-lipopolysaccharide (LPS) treatment conditions. Food intake and body weight (BW) were recorded and VAT mass and leptin mRNA levels were evaluated. Data indicated that MSG rats were lighter and displayed hypercorticosteronemia, hypophagia, adipoinsular axis hyperactivity, and enhanced VAT mass associated with an increased leptin gene expression. Interestingly, metformin-treated MSG rats corrected BW catch-up and counteracted VAT (mass and leptin mRNA level) and adipoinsular axis (basal and post-LPS) dysfunctions. Thus metformin treatment in MSG rats is able to correct several VAT and metabolic-endocrine dysfunctions. Our study suggests that a low-dose metformintherapy is effective to correct, at least in part, adipoinsular axis dysfunction in hypertrophic obese phenotypes, such as that of the human Cushing syndrome.

## 1. Introduction

Neonatal monosodium L-glutamate (MSG) administration induces morphological, behavioral, and neuroendocrine abnormalities such as growth disturbances, self-mutilation, hyperadiposity, and hypogonadism [1–4]. It has been assessed that catecholaminergic and peptidergic neuronal loss takes place in the retina and hypothalamic arcuate nucleus (ARC) [4–6]. As a consequence, body's energy balance [7–9], pituitary [10–12], and adrenal [13] activities become disrupted. A conspicuous effect of MSG-induced hypothalamic damage is an overall enhanced response of median eminence (ME) neuron terminals [10–12]. Thus, extensive brain damage has been ascribed to explaining neuroendocrine malfunctions in this model [14–16]. Acute inflammatory stress affects anterior pituitary hormone secretion by mechanisms involving different hypothalamic neuronal systems [17–21], already altered in the MSG animal model [4–6]. The ARC is a pivotal structure involved in the regulation of energy storage and expenditure [22], and both adipose tissue leptin secretion and hypothalamic leptin signaling system play key roles in maintaining homeostasis [22]. Among others, MSG rats are hyperleptinemic [23] due to hypertrophic adipose tissue (AT) mass expansion [24]. MSG rats are partly refractory to leptin inhibition of food intake and body weight gain [25], and overall leptin resistance seems to be directly dependent on enhanced glucocorticoid production [13, 26].

We previously reported that the adult male MSG rat displays neuroendocrine-immune dysfunction during the acute phase response of endotoxemia [27]. In the present study we explored in the MSG male rat model whether metformin treatment could be effective to ameliorate endocrine-metabolic-immune dysfunction, in both basal and post-lipopolysaccharide (LPS) administration conditions.

## 2. Material and Methods

### 2.1. Animals and Treatment

Adult male and female Sprague-Dawley rats were allowed to mate in colony cages in a light- (lights on from 07:00 to 19:00 h) and temperature- (22°C) controlled room. Rat chow and water were available* ad libitum*. Pregnant rats were transferred to individual cages. Beginning on day 2 after parturition, newborn pups were injected i.p. with either 4 mg/g body weight (BW) MSG (Sigma Chemical Co., St. Louis, MO) dissolved in sterile 0.9% NaCl or 10% NaCl (litter-mate controls; CTR) once every two days and up to day 10 of age [12]. Rats were weaned and sexed at 21 days of age, and male animals (CTR and MSG) were group-caged (3 rats per cage) and supplied with Purina chow and water* ad libitum*. Animals were left undisturbed until 59 days of age. CTR and MSG rats were used for experimentation on day 90 of age. MSG-injected animals were screened for effectiveness of treatment by macroscopic observation of degeneration of the optic nerves at the time of sacrifice and monitoring medial basal hypothalamic (MBH) levels of NPY mRNA. Animals were sacrificed according to protocols for animal use, in agreement with NIH Guidelines for care and use of experimental animals. All experimentation received approval from our institutional animal care committees.

### 2.2. Experimental Designs

On day 59 of age, rats were individually caged, and daily BW and food intake were recorded between days 60 and 90 of age. Experimental groups (*n* = 18 rats per group) were (i) CTR, (ii) MSG, and (iii) MSG animals receiving metformin (Craveri Lab., Argentina; at the dose 50 mg/Kg/day, dissolved in the drinking water, between days 60 and 90 of age; MSG + metformin) [28].

**Experiment 1 .**

Rats were sacrificed on the morning of the experimental day (90 days of age; 8 individuals per group) in nonfasting (basal) condition. Trunk blood was collected and plasma samples were kept frozen (−80°C) until assayed for glucose [27], nonesterified fatty acids (NEFA; Randox Laboratories Ltd., UK), triglycerides [27], corticosterone (B) [27], leptin [27], and insulin [27], by following the previously specific assays. Immediately after sacrifice, the MBHs were quickly dissected [21] and kept frozen (−80°C) until total RNA extraction. Thereafter, the visceral adipose tissue (VAT; abdominal pad) was dissected, weighed, and kept frozen (−80°C) until total RNA extraction. While the mRNA levels of neuropeptide Y (NPY) were evaluated in the MBH, those of leptin (LEP) in the VAT pad were measured.

**Experiment 2 .**

Rats were implanted, 48 hours before experimentation and under light ketamine anesthesia, with iv catheters and left undisturbed in individual cages, with food and water available* ad libitum*. On the morning (08:00 h) of the experimental day (90 days of age; 8 individuals per group), rats were bled before (between 2 and 5 min pretreatment; sample time zero) and 1, 2, 3, and 4 hours after i.v. administration of a sublethal dose of bacterial LPS (25 *μ*g/Kg; Sigma Chem. Co.) [29]. As described, blood samples taken were replaced by a similar volume of red blood cells resuspended in artificial plasma [29]. Plasma samples were split in aliquots and kept frozen (−80°C) until assayed for glucose [27] and triglycerides [27] concentrations.

### 2.3. Tissue RNA Isolation and qRT-PCR (Quantitative Real-Time PCR)

Total RNA was isolated from tissues (MBH blocks and VAT pads) by the single-step acid guanidinium isothiocyanate-phenol-chloroform extraction method (Trizol; Invitrogen, Life Tech., USA; Cat. number 15596-026) [30]. One *μ*g of total RNA was reverse-transcribed using random primers (250 ng) and Superscript III RNase HReverse Transcriptase (200 U/HL Invitrogen, Life Tech, USA; Cat number 18989-093). Two *μ*L of reverse transcription mix was amplified with 10 *μ*L of QuantiTect SYBRGreen PCR solution kit (Qiagen, Cat. number 204143), in the presence of 1 *μ*L of each specific primer (0.5 *μ*M final concentration), and revealed using a LightCycler Detection System (MJ MiniOpticon, Biorad). Primers (shown in alphabetical order in Table 1) were *β*-actin (ACTB), LEP, and neuropeptide Y (NPY). PCR efficiency was near to 100%. Threshold cycles (Ct) were measured in separate tubes, in duplicate. Identity and purity of the amplified product were checked by electrophoresis on agarose mini-gels and the melting curve was analyzed at the end of amplification. The differences between Cts were calculated in every sample for each gene of interest as follows: Ct gene of interest—Ct ACTB gene. Relative changes in the expression level of a specific gene (ΔΔCt) were calculated as ΔCt of the test group minus ΔCt of the CTR group and then expressed as 2^−ΔΔCt^.

### 2.4. Statistics

Data are expressed as mean ± SEM. Means were analyzed by two-way ANOVA (either with repeated measures or not), followed by the Student-Newman-Keuls test for comparison of different means. Results of tissue mRNA concentrations were analyzed by ANOVA and the nonparametric Mann-Whitney test [31].

## 3. Results

### 3.1. Rat Phenotype

**Figure 1:**
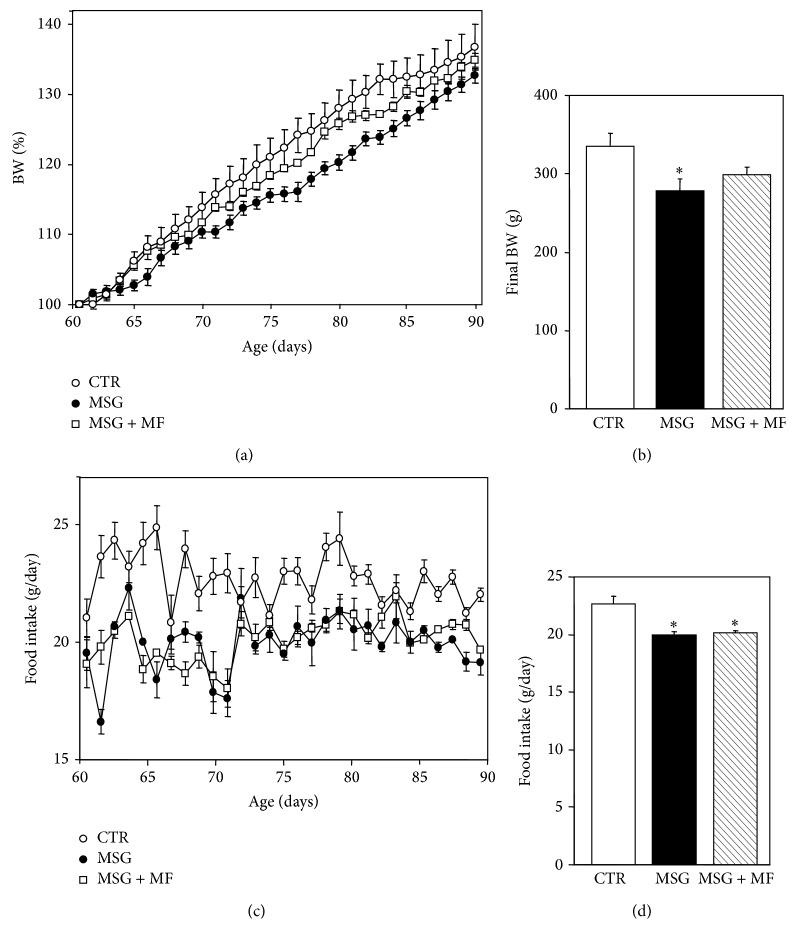
Animal growing curves drawn throughout ages 60 to 90 days (a) and body weight values recorded at age 90 days (b) in different groups of experimental animals. Daily food intake registered between 60 and 90 days of age (c) and the 30-day average of daily food intake and body weight values recorded at age 90 days (d) in CTR, MSG, and MSG + metformin (MSG + MF) rats. Values means ± SEM (*n* = 8 rats per group). ^∗^ *P* < 0.05 or less versus CTR values.

Departing (age 60 days) BW values already indicated that MSG rats were lighter than CTR rats (216.83 ± 7.15 versus 245.38 ± 9.28 g, resp.; *P* < 0.05). On this day, animals were individually caged, and MSG rats were allocated in two subgroups according to whether they were orally treated or were not treated with metformin (MSG + metformin and MSG rats, resp.). On the experimental day (90 days of age), MSG rats displayed significantly (*P* < 0.05) lower BWs than CTR rats; conversely, metformin-treated MSG rats (MSG + metformin) reached this day with indistinguishable BWs from those of CTR rats (Figure 1(b)). The slopes (expressed in percent of increase per day) of the 30-day growing curves (Figure 1(a)) indicated that MSG rats grew slower (1.13 ± 0.04) than CTR rats (1.35 ± 0.08) (*P* < 0.03); interestingly, the stunted growth displayed by MSG animals was fully corrected by metformin treatment (1.21 ± 0.02; *P* > 0.05 versus CTR values).

As depicted in Figure 1(c), MSG rats that were or were not treated with metformin displayed hypophagia when compared with CTR rats. Indeed, the daily rat food intake (expressed as the 30-day average, after recorded between ages 61 and 90 days) was significantly (*P* < 0.0001) lower in MSG rats, either treated or untreated with metformin, than in CTR animals (Figure 1(d)). The hypophagia characterizing MSG rats concords with the lower abundance of NPY mRNA in their MBHs. In fact, MBH NPY mRNA was significantly reduced in MSG rats, regardless whether or not they received metformin treatment (0.47 ± 0.22 versus 1.02 ± 0.37 arbitrary units; *P* < 0.03).

### 3.2. Effect of Metformin Treatment on Visceral Adipose Tissue Mass and Leptin Gene Expression

**Figure 2:**
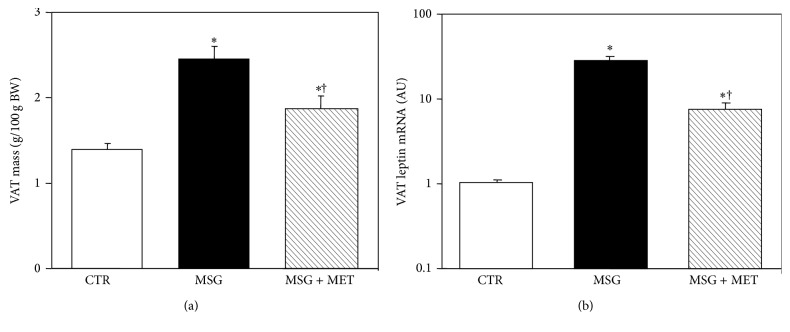
Visceral adipose tissue (VAT) mass (a) and mRNA levels (in arbitrary units, AU) of leptin (b) in 90-day-old CTR and MSG-damaged male rats, either treated or untreated with metformin (MSG + MF). Values are means ± SEM (*n* = 4-5 pads per group). ^†^ *P* < 0.05 versus MSG values.  ^∗^ *P* < 0.05 versus CTR values.

The VAT pad mass was higher (*P* < 0.05) in MSG (8.02 ± 0.41 g) than in CTR (4.03 ± 0.22 g) rats, and metformin treatment (MSG + metformin rats) was effective to significantly (*P* < 0.05 versus MSG and CTR group-values) reduce this parameter (6.02 ± 0.71 g). Indeed, relative to individual BW values, the VAT pad mass was 1.8-fold (*P* < 0.01) higher in MSG than in CTR rats, and metformin treatment in MSG rats (MSG + metformin) was able to significantly (*P* < 0.05 versus MSG values) reduce VAT mass, although mass values remained significantly (*P* < 0.05) higher than in CTR animals (Figure 2(a)).

Figure 2(b) shows the results of VAT mRNA levels of leptin in different groups. As depicted, VAT leptin mRNA concentration was severalfold higher in MSG than in CTR rats (*P* < 0.001). Interestingly, metformin treatment in MSG rats resulted in a significant (*P* < 0.01) reduction in the expression levels of the leptin gene in their VAT pads, although values remained higher (*P* < 0.02) than in CTR rats.

### 3.3. Peripheral Biomarkers

MSG animals studied in the basal (nonfasting) condition displayed an expected hypercorticosteronemia (13.92 ± 1.75 and 12.03 ± 1.57 *μ*g/dL in MSG and MSG + metformin rats, resp., versus 6.38 ± 1.26 *μ*g/dL in CRT rats; *P* < 0.05).

**Figure 3:**
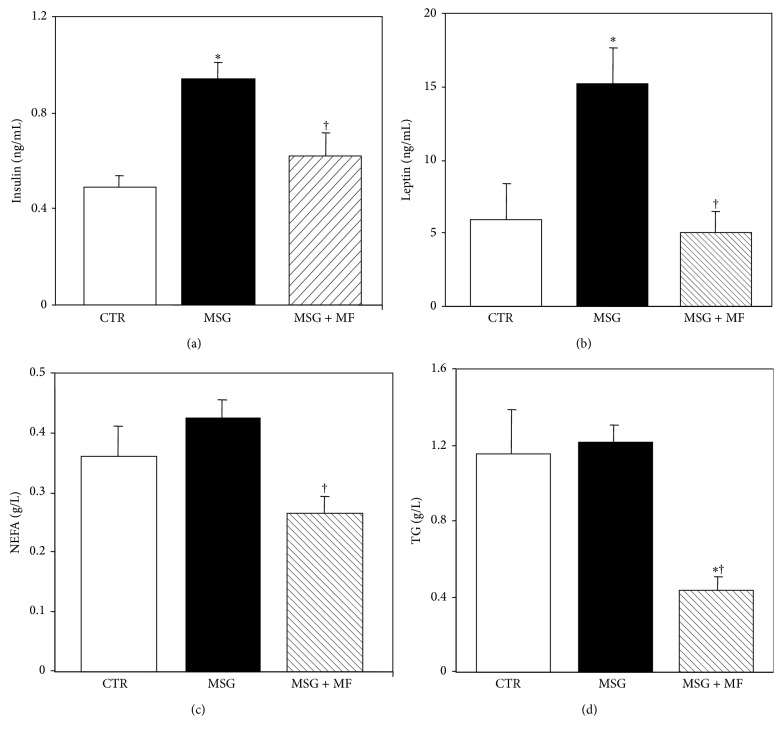
Circulating levels of markers of the adipoinsular axis function: insulin (a), leptin (b), nonesterified fatty acids (NEFA) (c), and triglycerides (TG) (d), in normal (CTR), MSG, and metformin-treated MSG (MSG + MF) male rats at 90 days of age. Values are means ± SEM (*n* = 8 rats per group). ^†^ *P* < 0.05 versus MSG values. ^∗^ *P* < 0.05 versus CTR values.

Analysis of the adipoinsular axis activity indicated that although glycemia was similar in all groups studied (101 ± 7, 107 ± 4, and 102 ± 5 mg/dL in CTR, MSG, and MSG + metformin groups, resp.), significantly (*P* < 0.05 versus CTR values) higher insulinemia was noticed in MSG than in CTR rats (Figure 3(a)). Interestingly, metformin treatment in MSG rats was able to significantly (*P* < 0.05 versus MSG group-values) reduce insulinemia, its values being now similar to those displayed by CTR animals (Figure 3(a)). As expected [27], MSG rats displayed severe hyperleptinemia (*P* < 0.05 versus CTR values) (Figure 3(b)); however, this adipose tissue dysfunction characterizing MSG rats was fully (*P* < 0.05 versus MSG values) abolished by metformin treatment (Figure 3(b)). Finally, MSG rats displayed normal basal NEFA and TG circulating levels (Figures 3(c) and 3(d), resp.), and metformin treatment in MSG rats significantly (*P* < 0.05) reduced plasma lipids levels (Figures 3(c) and 3(d)).

### 3.4. Glycemic and Triglyceridemic Profile throughout Acute Endotoxemia

**Figure 4:**
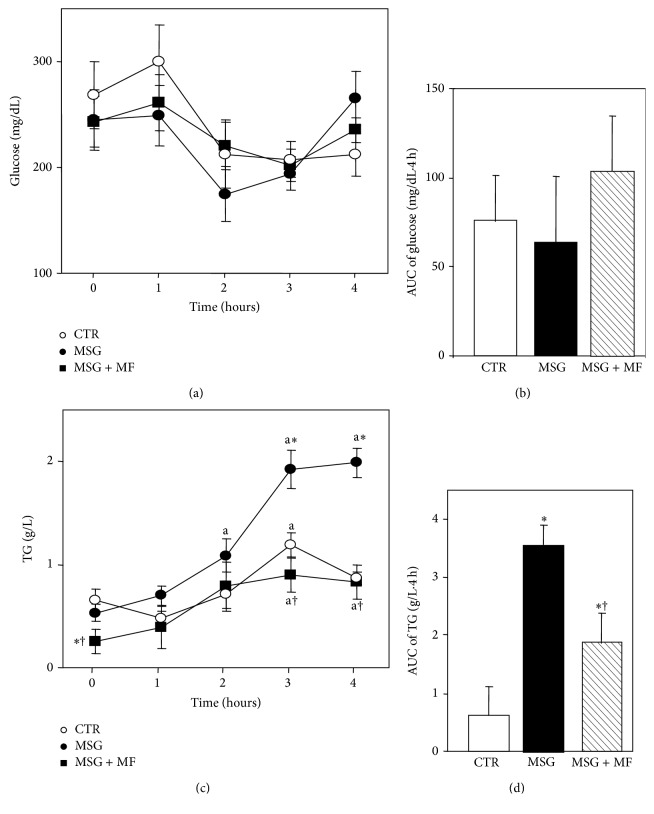
Peripheral levels of glucose (a) and triglycerides (c) before (time zero hours) and several hours after i.v. LPS (25 mg/Kg BW) treatment in different groups of rats. The area under the curve (AUC) of glycemia (b) and triglyceridemia (d) throughout endotoxemia is also displayed. Values are means ± SEM (*n* = 8 rats per group). ^a^ *P* < 0.05 versus time 0 values in the same group. ^∗^ *P* < 0.05 versus CTR values for a similar time. ^†^ *P* < 0.05 versus MSG values for a similar time.

Figure 4 shows the results of circulating glucose and triglycerides concentrations before (sample time 0) and several times (1–4 h) after endotoxemia. LPS i.v. administration did not induce any significant hypoglycemia in neither experimental group. Indeed, as depicted, circulating glucose levels (Figure 4(a)) were similar among groups in different times examined, and all groups displayed no significant time-dependent changes in plasma glucose concentrations when comparing values from the AUC of glycemia (Figure 4(b)).

Although basal triglyceridemia (Figure 4(c)) was similar in CTR and MSG groups, a significantly (*P* < 0.05) decreased basal lipid concentration was shown in MSG + metformin rats. LPS administration significantly (*P* < 0.05 versus respective time 0 values) enhanced triglyceridemia in CTR rats for the 3 h after LPS, thereafter returning to basal levels by the end of the test. Conversely, in MSG rats, plasma lipid levels were already significantly (*P* < 0.05 versus respective time 0 values) higher than the 2 h after LPS, and this effect lasted until the end of the experimental design (4 h). Interestingly, metformin treatment in MSG rats delayed the enhancement in plasma lipid concentration up to a similar time to that occurring in CTR rats (3 h); however, these rats did not recover their basal TG levels by the end of the test. Moreover, the AUC of triglycerides values was significantly (*P* < 0.01) higher in MSG than in CTR rats (Figure 4(d)). Interestingly, metformin treatment in MSG rats was able to partially prevent the enhanced lipidic response to LPS injection, as depicted by the AUC values of peripheral TG levels (Figure 4(d)).

## 4. Discussion

Our study indicates that MSG-induced neonatal hypothalamic damage resulted in the alteration of endocrine-metabolic function at the adult age and, importantly, that this multidysfunction can be improved by the treatment with a very low dose of metformin.

It should be stressed that although several dysfunctions in the adult male MSG rat, a phenotype of hypothalamic obesity, have been extensively revisited [1–13], only few studies have been focused on the metabolic improvement induced by metformin treatment in these animals. Indeed, data from previous studies performed in MSG rats treated with a high daily metformin dose (300–500 mg/Kg BW) [32–34] indicate that the treatment improves glucose metabolism [32–34], blood pressure [34], and cardiovascular function [31, 32].

The analysis of our animals' phenotypes indicated that, although all MSG rats remained hypophagic, their overall stunted growth (growing curve, low body weight, and large adiposity) was significantly ameliorated by metformin treatment. Thus, such an improvement is clearly indicative for the occurrence of favorable, metformin-dependent, metabolic-endocrine changes in MSG animals. Indeed, it has been reported that metformin treatment in high-fructose fed rats resulted in being able to reduce intra-abdominal adipose tissue mass by activating local sympathetic activity at the retroperitoneal adipose tissue level [35]. Taken into account that the MSG rat is characterized by an impaired sympathetic [36] and adrenal [37] catecholamine production, this metformin effect could highly contribute to reducing VAT mass by its increasing effect in sympathetic activity [35]. Moreover, and agreeing with our findings, metformin treatment in high-fat fed rats is able to improve metabolism and to reduce body fat mass, without affecting food intake and body weight [38].

In this regard, the adiposity dysfunction, namely that developed at the VAT level, in MSG rats was highly improved by the oral treatment of animals with a very low dose of metformin. Indeed, the high peripheral levels of insulin and leptin in the nonfasting condition (two metabolically different paths belonging to the adipoinsular axis) in MSG rats were fully corrected by metformin treatment. In addition, we have addressed that metformin treatment in MSG animals also was able to improve VAT dysfunction, such as tissue mass and leptin mRNA concentration. Accordingly, we have recently addressed that the treatment with enhancers of insulin activity, although in a model of diet-induced hyperadiposity, was effective in preventing hyperinsulinemia, dyslipidemia, hypertrophic expansion of abdominal adipose tissue mass, and adipocyte leptin mRNA overexpression, whose mechanisms were mainly mediated by the improvement of both IRS-1 and IRS-2 functionalities after metformin [39].

Regarding the lipidic metabolism in MSG rats, although there are some discrepancies among our data on unmodified peripheral TG concentrations, it must be mentioned that other researchers found enhanced basal TG levels where they have taken those samples at 8 or more hours after food was withdrawn (fasting condition) [40, 41]. When analyzing peripheral TG levels in response to LPS in MSG rats, it has been shown that MSG rats, over development, display reduced lipolytic and enhanced lipogenic activities [42], thus indicating that after the allostatic load (LPS injection) the balance of activities strongly support for the enhanced MSG AUC of this lipid [43, 44]. Moreover, other authors reported that metformin treatment in either high lipid- or high fructose-fed rats was able to prevent the development of oxidative stress and thus hyperlipidemia/-insulinemia [45, 46]. Indeed, a direct metformin reducing effect on NEFA production by isolated adipocytes has also been detected [47]. Interestingly, in the present experiments we found that, despite no changes in glycemia, the increased LPS-induced triglycerides secretion in plasma in MSG rats was also fully abolished by metformin treatment. It has been reported that metformin could reduce peripheral dyslipidemia by an indirect mechanism, related to diminished synthesis and enhanced clearance of VLDL particles [48], the main TG transporter in the circulation.

In relation with the intriguing lack of a peripheral glucose response, we previously found an increase in peripheral insulin levels after LPS treatment in rats [27]. Moreover, LPS treatment in MSG rats enhances also the secretion of immune system-derived cytokines and pancreatic insulin, and as a counterregulatory signal, glucagon; thus, the ratio of peripheral insulin : glucagon concentrations remains similar to that displayed by LPS-injected normal rats [27]. Moreover, animals under endotoxemia also develop an increase in glucocorticoid production that precedes that of insulin [27]; thus the enhanced glucocorticoid endogenous environment could be cooperating for maintaining glucose homeostasis [49], a phenomenon vital for survival during endotoxic shock.

Regarding the adipoinsular axis response to LPS, it is well known that in normal rats this stimulus enhances the secretion of both leptin [29] and insulin [50], and we previously found that in MSG rats such a response is clearly exacerbated [27]. Moreover, hyperleptinemic MSG rats also displayed basal hyperinsulinemia, agreeing with data from previous* in vivo* studies from our laboratory [27, 51] and with* in vitro* studies suggesting that in MSG rats the parasympathetic regulation of pancreatic activity is increased [52]. Interestingly, metformin treatment in MSG was able to fully reverse hyperinsulinemia and hyperleptinemia in the basal condition. The mechanism whereby this metformin effect takes place has been ascribed to its intrinsic inhibition of liver glucose production (directly related to insulinemia) and to a VAT mass lowering effect. Indeed, metformin administration in MSG did also result in a significant decrease in leptin mRNA abundance in their VAT pads. The decrease in VAT mass and the reduced insulinemia and leptinemia induced by metformin treatment in MSG rats also could benefit the LPS-induced dyslipidemia.

In conclusion, our study demonstrates that many of the host's defense mechanisms are deeply disturbed in MSG rats and are of relevance, so that a very low oral dose of metformin during one month is able to ameliorate this rat phenotype. It is largely accepted that the main site of MSG action is the ARC and that not only NPYergic [26] but also other hypothalamic neuronal activities are impaired in MSG rats [43, 53]. Nevertheless, despite a lack of effect of metformin on food intake, treating MSG rats with a very low dose of this compound did result in a significant improvement in body weight catch-up (displaced to a lower fat mass) and several metabolic-endocrine dysfunctions, namely those of the adipoinsular axis. Thus, our study adds more evidence to the efficacy of metformin treatment for lowering cardiovascular risk in hyperadipose phenotypes characterized by an excess of endogenous glucocorticoid, as occurs in human Cushing's syndrome [44]. It remains to be further explored whether a larger metformin dose could be effective to correct (partly/fully) hypercorticosteroidism in MSG rats.

## Figures and Tables

**Table 1:** Rat-specific primers (in alphabetical order) designed and used for real-time PCR analyses.

		GBAN	bp
ACTB	se, 5′-AGCCATGTACGTAGCCATCC-3′	NM_031144	115
	as, 5′-ACCCTCATAGATGGGCACAG-3′		

LEP	se, 5′-GAGACCTCCTCCATCTGCTG-3′	NM_013076	192
	as, 5′-CTCAGCATTCAGGGCTAAGG-3′		

NPY	se, 5′-TACTCCGCTCTGCGACACTA-3′	NM_012614	115
	as, 5′-GGGCATTTTCTGTGCTTTCT-3′		

se: sense; as: antisense; GBAN: GenBank Accession Number; amplicon length, in bp.
